# Immune Dysregulation in Cancer Patients Undergoing Immune Checkpoint Inhibitor Treatment and Potential Predictive Strategies for Future Clinical Practice

**DOI:** 10.3389/fonc.2018.00080

**Published:** 2018-03-22

**Authors:** Ronald Anderson, Bernardo L. Rapoport

**Affiliations:** ^1^Department of Immunology, University of Pretoria, Pretoria, South Africa; ^2^Institute for Cellular and Molecular Medicine, Faculty of Health Sciences, University of Pretoria, Pretoria, South Africa; ^3^The Medical Oncology Centre of Rosebank, Johannesburg, South Africa

**Keywords:** iomarkers, CTLA-4, enterocolitis, interleukin-17, monoclonal antibodies, programmed cell-death-1, T helper 17 cells

## Abstract

Realization of the full potential of immune checkpoint inhibitor-targeted onco-immunotherapy is largely dependent on overcoming the obstacles presented by the resistance of some cancers, as well as on reducing the high frequency of immune-related adverse events (IRAEs) associated with this type of immunotherapy. With the exception of combining therapeutic monoclonal antibodies, which target different types of immune checkpoint inhibitory molecules, progress in respect of improving therapeutic efficacy has been somewhat limited to date. Likewise, the identification of strategies to predict and monitor the development of IRAEs has also met with limited success due, at least in part, to lack of insight into mechanisms of immunopathogenesis. Accordingly, considerable effort is currently being devoted to the identification and evaluation of strategies which address both of these concerns and it is these issues which represent the major focus of the current review, particularly those which may be predictive of development of IRAEs. Following an introductory section, this review briefly covers those immune checkpoint inhibitors currently approved for clinical application, as well as more recently identified immune checkpoint inhibitory molecules, which may serve as future therapeutic targets. The remaining and more extensive sections represent overviews of: (i) putative strategies which may improve the therapeutic efficacy of immune checkpoint inhibitors; (ii) recent insights into the immunopathogenesis of IRAEs, most prominently enterocolitis; and (iii) strategies, mostly unexplored, which may be predictive of development of IRAEs.

## Introduction

Genetic engineering combined with other sophisticated molecular and immunological technologies has greatly enhanced the range of clinical applications and efficacy of monoclonal antibody (MAb)-based immunotherapeutic strategies with onco-immunotherapy being possibly the most prominent beneficiary (1, 2). Notwithstanding the refinement of cell-based immunotherapies, the development of fully humanized and, in particular, fully human MAbs, targeted against immune checkpoint inhibitory molecules expressed on tumor-infiltrating cells of the innate and adaptive immune systems, as well as their ligands expressed on tumor cells, has transformed the promise and practice of onco-immunotherapy (1, 2).

Fully humanized MAbs retain antigen recognition immune complementarity-determining regions (CDRs) fused with genetically modified V regions of human immunoglobulin (Ig) mostly of the IgG1 and IgG4 subclasses to generate the functional MAb (3). Fully human MAbs, which have no murine sequences, are generated by phage display or mostly by using transgenic mouse technology, enabling replacement of murine Ig genes with functional human loci (3). However, these engineered therapeutic MAbs, even some which are fully human, may retain immunogenicity in their CDR regions, which may be eliminated by minor (“up to two”) amino acid substitutions in the setting of retention of bioactivity (3). MAbs of the IgG1 and IgG4 subclasses are generally preferred because of their relatively long half-lives of ≥21 days, while the lack of complement fixation activity of IgG4 is an added advantage. In the case of human/humanized MAbs of other IgG subclasses, complement fixation properties are attenuated by the implementation of numerous mutations in the CH2 region of the antibody molecule, with the majority of all therapeutic MAbs currently licensed or in development being of the IgG1 subclass (4).

These innovations in the design and production of MAbs have not only improved the efficacy and safety of MAb-based therapies for various types of cancer and autoimmune disease, but as a result of an extended elimination time they have also reduced the frequency of administration. Nonetheless, re-directing a finely tuned immune system to achieve therapeutic benefit remains an intricate, albeit a challenging and exciting, science. Consequently, despite the favorable risk:benefit of MAb-based therapy in advanced malignant diseases, there remains an ongoing need for careful monitoring of patients in the setting of an awareness on the part of the attending clinician of the potential for development of adverse immunological reactions. This concern is clearly underscored by an earlier experience with the humanized IgG4 Mab known as TGN1412 (5).

TGN1412, also known as CD28 SuperMAB/TAB 08, promotes antigen-independent activation and expansion of T cells *via* its agonistic interaction with the co-stimulatory molecule CD28. TGN1412 was developed primarily for the immunotherapy of T cell primary immunodeficiency disorders, as well as B cell chronic lymphocytic leukemia and rheumatoid arthritis (RA), the latter because of the preferential expansion of Th2 cells and CD4^+^, CD25^+^ regulatory T cells (Tregs) induced by a murine counterpart antibody, which had demonstrated no indication of immunological hyperreactivity during pre-clinical assessment (5). Progression of development to phase 1 clinical evaluation proved, however, to be calamitous. A single intravenous infusion of TGN1412 administered to six young healthy adult male volunteers resulted in an abrupt (within 90 min) systemic inflammatory response associated with dramatic, transient elevations in the levels of the circulating pro-inflammatory cytokines, interleukin (IL)-1β, IL-2, IL-6, IL-8, tumor necrosis factor (TNF)-α, and interferon (IFN)-γ (5). Given the lack of correlation between the immunomodulatory activities of human/humanized and murine CD28 targeted MAbs, these findings clearly underscore the unpredictable outcome of therapeutic strategies based on fine tuning of the human system. This may be of particular importance in disease settings in which the equilibrium of the immune system is already perturbed due to co-existent, sub-clinical inflammatory disorders.

Despite these concerns, the field of onco-immunotherapy has burgeoned in very recent times due in large part to the development of both humanized and human MAbs which neutralize various types of immune checkpoint inhibitory molecules. Although continuing to expand rapidly with the development of novel MAbs targeted against an increasing range of negative immune checkpoint molecules, many of which are currently undergoing phase I–III clinical trials (2), the majority of published clinical studies have evaluated the therapeutic potential of those developed and approved at an earlier stage, between 2011 and 2014, which target cytotoxic T-lymphocyte-associated-4 (CTLA-4; CD152), programmed cell-death-1 (PD-1; CD279) and its counter ligands PD-L1 (CD274) and PD-L2 (CD273). It is now well recognized that immune checkpoint inhibitory molecules are inextricably involved in mediating an immunosuppressive milieu which promotes tumorigenesis and tumor progression, with the two most studied mechanisms being those involving CTLA-4 and PD-1 (1, 2). Over-expression of CTLA-4 by Tregs in particular subverts T cell activation and expansion, while interaction of PD-1 on effector T cells compromises anti-tumor cytokine production and cytotoxicity. Blockade of CTLA-4- and PD-1-mediated immunosuppression promotes restoration of anti-tumor immune function, but if excessive may also pose the risk of tissue damage and autoimmunity (1, 2).

Although the clinical response rates (tumor regression) of these agents are relatively low, being around 20% for monotherapy and somewhat higher for combination therapy (6–10), this must be balanced against the fact that treatment with these agents is associated with durable remissions and long-term survival in patients with metastatic malignant melanoma, non-small cell lung cancer (NSCLC), bladder cancer, and other types of tumor. In this new era of personalized medicine, the utilization of biomarkers has emerged as an essential concept in patients undergoing anti-PD-1/anti-PDL-1 therapy. In this context, it has recently been shown that patients with metastatic NSCLC with expression of PD-L1 on at least 50% of tumor cells, treatment with pembrolizumab (an anti-PD-1 antibody) is associated with considerably longer progression-free and overall survival, as well as with fewer adverse events compared with platinum-based chemotherapy (11).

In addition to onco-immunotherapy, there is also increasing interest in the use of these various immunostimulatory checkpoint MAbs in the adjuvant therapy of both acute (sepsis) and chronic infectious diseases (12–14), particularly therapy-intransigent tuberculosis and HIV/AIDS (10, 12, 13), as well as primary and secondary immunodeficiency disorders, and hepatitis B and C virus-associated hepatocellular carcinoma (15).

Despite the undoubted success of, and enthusiasm for, MAb-mediated neutralization of immune checkpoint inhibitors in the onco-immunotherapy of various types of advanced cancer, the full therapeutic efficacy of these agents remains to be realized. Notwithstanding the occurrence of common, albeit less serious side-effects, including cough, fatigue, loss of appetite, nausea, skin rash, and itching, it is the resistance of some cancers (8), together with the very high frequency of sometimes serious, immune-related adverse events (IRAEs), which represent the most significant obstacles confronting the success of immune checkpoint inhibitor therapy (16).

The remaining sections of this review are focused on brief considerations of CTLA-4- and PD-1/PD-L1-targeted MAbs currently in clinical use, as well as more recently identified negative immune checkpoint inhibitor molecules, which may serve as future therapeutic targets. The subsequent and more extensive sections are focused on strategies which may improve the efficacy of anti-cancer immune checkpoint inhibitor therapy, followed by overviews, firstly of putative mechanisms of immunopathogenesis of IRAEs, most prominently CTLA-4 blockade-associated enterocolitis and, finally, strategies, both recognized and proposed, which may enable early identification of those patients with advanced cancer who may be at highest risk for development of IRAEs.

## Immune Checkpoint Inhibitor MAbs Approved for Clinical Application

Monoclonal antibodies currently approved for clinical application in onco-immunotherapy include: (i) ipilimumab (the first approved for clinical application in 2011), while tremelimumab is in the advanced stages of clinical evaluation, both of which target CTLA-4; (ii) the PD-1 antagonists, nivolumab, and pembrolizumab; and (iii) the PD-L1 inhibitors, avelumab, atezolizumab, and durvalumab (17–22). The major characteristics and clinical applications of these therapeutic MAbs are summarized in Table 1.

**Table 1:** Currently approved immune checkpoint inhibitory monoclonal antibodies and their clinical applications in onco-immunotherapy.

Drug	Immune checkpoint target	Indication
Ipilimumab^a^	CTLA-4	Unresectable metastatic melanoma In combination with nivolumab for unresectable or metastatic melanoma Adjuvant therapy with stage III melanoma
Pembrolizumab	PD-1	Melanoma advanced or unresectable Metastatic NSCLC with PDL-1 expression Metastatic NSCLC with progression on or after platinum therapy Metastatic NSCLC in combination with pemetrexed and carboplatin, as first-line treatment of patients with metastatic non-squamous NSCLC Recurrent SCCHN Classical Hodgkin’s lymphoma (cHL) for the treatment of adult and pediatric patients with refractory cHL, or who have relapsed after three or more prior lines of therapy Urothelial carcinoma for the treatment of patients with locally advanced or metastatic urothelial carcinoma who are not eligible for cisplatin-containing chemotherapy Urothelial carcinoma for the treatment of patients with locally advanced or metastatic urothelial carcinoma who have disease progression during or following platinum-containing chemotherapy or within 12 months of neoadjuvant or adjuvant treatment with platinum-containing chemotherapy Microsatellite instability-high cancer (MSI-H) for the treatment of adult and pediatric patients with unresectable or metastatic, MSI-H or mismatch-repair-deficient solid tumors that have progressed following prior treatment and who have no satisfactory alternative treatment options, or colorectal cancer that has progressed following treatment with a fluoropyrimidine, oxaliplatin, and irinotecan. Gastric cancer for the treatment of patients with recurrent locally advanced or metastatic gastric or gastroesophageal junction adenocarcinoma whose tumors express PD-L1 as determined by an FDA-approved test, with disease progression on or after two or more prior lines of therapy including fluoropyrimidine- and platinum-containing chemotherapy and if appropriate, HER2/neu-targeted therapy
Nivolumab	PD-1	Unresectable or metastatic melanoma with progression after ipilimumab or BRAF inhibitor if BRAF V600 mutant In combination with ipilimumab for unresectable or metastatic melanoma NSCLC with progression on or after platinum therapy Metastatic RCC after prior anti-angiogenic therapy cHL: recurrent Recurrent or metastatic squamous cell carcinoma of the head and neck Locally advanced or metastatic urothelial carcinoma MSI-H or mismatch-repair-deficient metastatic colorectal cancer Hepatocellular carcinoma
Atezolizumab	PDL-1	NSCLC with progression on or after platinum therapy Urolthelial carcinoma with progression on or after platinum therapy
Durvalumab	PDL-1	Locally advanced or metastatic urothelial carcinoma who have disease progression during or following platinum-containing chemotherapy Locally advanced or metastatic urothelial carcinoma who have disease progression within 12 months of neoadjuvant or adjuvant treatment with platinum-containing chemotherapy
Avelumab	PDL-1	Indicated for the treatment of adults and pediatric patients 12 years and older with metastatic Merkel cell carcinoma

*^a^Data from Ref. (17–22)*.

Although these MAbs have been used individually in onco-immunotherapy, it is combinations of MAbs, which target different immune checkpoint inhibitors, particularly CTLA-4 and PD-1 using ipilimumab and nivolumab, respectively, which have been shown to be most effective in prolonging progression-free survival and overall response rates in patients with metastatic/unresectable melanoma and other types of cancer. Additionally, the use of combination therapy with immune checkpoint inhibitors has been associated with substantial increases in the frequency of IrAEs and treatment discontinuations (7, 9, 23, 24).

## Alternative Negative Immune Checkpoint Molecules Which May Serve as Targets for Immunostimulatory MAbs

In addition to CTLA-4, PD-1, PD-L1/L2, as well as indoleamine 2,3-dioxygenase produced mainly by plasmacytoid dendritic cells and killer Ig-like receptor expressed on natural killer cells (8), more recently identified inhibitory immune checkpoint molecules expressed on T cells, which are potential targets for onco-immunotherapy and which are currently undergoing early clinical evaluation include:

- T cell Ig domain and mucin protein 3 (CD366), which appears to interact with galectin-9, as well as several other ligands on tumor cells (25).
- Lymphocyte activation gene-3 (CD223), which downregulates T cell activation *via* interaction with major histocompatibility class II molecules (25).
- V-domain Ig suppressor of T cell activation, which downregulates T cell proliferation and cytokine production *via* interaction with a putative ligand(s), which remains to be identified (26–28).

Other immune checkpoint molecules which show early promise as potential targets for MAb-mediated immunotherapy include T cell immunoreceptor with Ig and ITIM domains, B and T lymphocyte attenuator (CD272), and V-set Ig domain containing 4 (2, 8, 25, 26).

## Strategies Which May Improve the Therapeutic Efficacy of Negative Immune Checkpoint Molecule-Targeted Immunotherapy

This important field of translational research is the subject of a recent, extensive review by Greil et al. (2).

### Pre-Therapy Detection of Immune Checkpoint Inhibitory Molecules and Their Ligands on Intra-Tumoral T Cells and Tumor Cells

One of the most favored, but not entirely proven strategies, involves the pre-therapy detection of expression of inhibitory immune checkpoint molecules on intra-tumoral T cells and/or their ligands on tumor cells (2). In the context of predictive personalized immunotherapy, it is noteworthy that the expression of PD-L1 in a range of different types of tumor biopsies (melanoma, NSCLC, renal cell carcinoma, colon carcinoma, bladder carcinoma, and hematologic malignancies) is predictive of a favorable outcome to PD-1/PD-L1-targeted therapy (29). Alternative predictive strategies include measurement of expression of PD-1 or CTLA-4 on circulating T cells, as well as the levels of soluble immune checkpoint inhibitors and/or their ligands by serological testing and/or detection of their RNA transcripts (30–34).

### Augmentation of Tumor Immunogenicity

Other strategies include measurement of the tumor mutational burden as an independent predictor of both tumor immunogenicity and the response to negative immune checkpoint blockade (2, 35). Greil et al. in their recent review also mention the potential of modulation of activated members of the apolipoprotein B mRNA editing enzyme catalytic polypeptide-like gene family members as a strategy to increase tumor neoantigeniticy (2). The same authors also advocate broadening of the T cell receptor repertoire *via* “therapeutic strategies aimed at reactivating or boosting the host anti-tumor immune response” to improve the response to checkpoint inhibitors (2).

### Pre-Therapy Detection of Immunosuppressive and Immunostimulatory Cytokines

Importantly, the efficacy of inhibitory immune checkpoint molecule-targeted therapy may be countered by the co-existence of alternative tumor-related immunosuppressive mechanisms. Foremost among these is the immunosuppressive cytokine, transforming growth factor-β (TGF-β), which aside from negating anti-tumor host defenses, can also promote tumorigenesis, metastasis, and chemoresistance (36). In this context, it is noteworthy that MAb-mediated neutralization of two of the three isoforms of TGF-β, *viz*. TGF-β1 and TGF-β2, was found to potentiate both vaccine and PD-1-targeted immunotherapy in a murine model of experimental cancer therapy (37). In the clinical context, a “dichotomized risk score” combining baseline levels of circulating TGF-β1 and another immunosuppressive cytokine *viz*. IL-10, but not TGF-β1 alone, was predictive of decreased progression-free survival in ipilimumab-treated patients with advanced melanoma (HR = 2.66; *P* = 0.035) (38). Although the findings of this small, but under-powered study may be found to be important in the future, the role of IL-10 and TGF-β1 in this context will need to be confirmed in larger, adequately powered prospective studies. In addition, while TGF-β is well recognized as a probable key determinant of the therapeutic efficacy of immune checkpoint inhibitors (39), adjunctive immunological or pharmacological targeting of this cytokine must be tempered by an awareness of the attendant risk of cumulative immune dysregulation. Nonetheless, prior detection of elevated levels of circulating TGF-β may identify a sub-group of patients with advanced metastatic cancer who may experience added benefit from dual immune checkpoint inhibitor-/TGF-β-targeted immunotherapy (40, 41).

On the other hand, it has been reported that elevations in the pre-therapy serum concentrations of the cytokines IFN-γ (*P* < 0.0001), IL-6 (*P* < 0.0007), and IL-10 (*P* < 0.0001) are predictive of treatment efficacy in patients with advanced melanoma receiving nivolumab (42). The findings in relation to IL-10 appear, however, to contradict those described in the above in the study reported by Tarhini et al. (38).

### Alterations in the Numbers of Circulating Leukocytes and Leukocyte Subsets, Soluble CD25, and Lactate Dehydrogenase

As recently reviewed in detail by Manson et al., additional biomarkers which appear to be associated with favorable responses particularly to ipilimumab in the setting of advanced melanoma, and possibly nivolumab therapy of NSCLC, include: (i) higher pre-therapy circulating lymphocyte counts, as well as rising lymphocyte counts during therapy; (ii) elevated neutrophil:lymphocyte and platelet:lymphocyte ratios pre-therapy; (iii) a declining neutrophil:lymphocyte ratio during therapy; (iv) low numbers of circulating eosinophils pre-therapy; (v) high numbers of CD16^+^ monocytes pre-therapy; (vi) increasing numbers of circulating CD4^+^ T cells with high-level expression of ICOS (inducible T cell costimulator, a member of the CD28 family); (vii) low levels of circulating soluble CD25, an antagonist of IL-2; and (viii) a high-baseline level of lactate dehydrogenase (43–45).

### Gene Profiling of Circulating Leukocytes

Very recently, Friedlander et al. reported on the potential utility of a blood RNA transcript-based model targeting 169 genes to predict the clinical response of stage IV melanoma patients to tremelimumab in two independent studies. In the first of these, to which treatment-naïve patients (*n* = 210) were recruited (“discovery data set”), a 15—gene signature was identified which predicted both an objective clinical response and 1-year survival after treatment (46). The genes identified were categorized as either “predictor” (*n* = 9) or “enhancer,” the latter being found to enhance the performance of the “predictors” (46). Proteins encoded by the “predictor” genes were: cyclin-dependent kinase 2; cyclin-dependent kinase inhibitor 2A; dipeptidyl peptidase 4; erb-b2 receptor tyrosine kinase 2; ICOS; integrin subunit α4; a member of the *N*-acetylglucosaminyltransferase family (LARGE); NGFI-A-binding protein 2; and *N*-Ras GTPase. Those in the “enhancer” category were: ADAM metallopeptidase domain 17; HLA-DR α-chain; Myc transcription factor; RHoC, a Rac sub-family GTPase; TGF-β1; and tissue inhibitor of matrix metalloproteinase 2 (46). These findings were validated in a second study to which advanced melanoma patients (*n* = 150) who received tremelimumab after chemotherapy were recruited (46).

### HLA Typing

Although the potential utility of HLA typing in predicting therapeutic responses to immune checkpoint inhibitors is largely unexplored, in this context a recent report by Ishida et al. is noteworthy (47). These investigators, using a DNA-based HLA typing procedure, albeit in a relatively small group (*n* = 69) of Japanese melanoma patients, reported a statistically significant association between positivity for the HLA-A*26 allele and responsiveness to therapy with nivolumab (OR = 4.93, *P* = 0.028) (48). The authors concede, however, that in addition to the small number of patients recruited to their study that confirmation of their findings in different population groups is necessary, while alluding to an earlier study in which HLA typing had no predictive value in lung cancer patients treated with pembrolizumab (48).

### Smoking History

Smoking history may also be predictive of response to therapy. In this context, smoking has been reported to be associated with increased expression of PD-1 on circulating CD4^+^ and CD8^+^ T cells from apparently healthy young smokers and, in particular, HIV-infected smokers, relative to groups of matched non-smokers, as well as with upregulated expression of CTLA-4 on CD4^+^ T cells (49). It is therefore noteworthy that Calles et al. recently reported detection of PD-L1 expression in *KRAS*-mutant NSCLC specimens from 44, 20, and 13% of current smokers, former smokers, and never-smokers, respectively (*P* = 0.03) (50), which has been reported to correlate with a favorable response to PD-1/PD-L1-targeted therapy (29, 51). Smoking history, particularly current smoking, therefore appears to be a determinant of a favorable outcome of PD-1/PD-L1 therapy in NSCLC, possibly related to the suppressive effects of smoking on pulmonary immune function (52), as well as to increased tumor mutational load and neoantigenicity mediated by smoke-derived carcinogens.

Although this section of the review has highlighted a range of potential predictors of favorable responses to immune checkpoint inhibitor therapy, the most practicable of which are summarized in Table 2, widespread implementation of a number of these may often be difficult due to cost and/or lack of access of oncologists to sophisticated, molecular immunology capability.

**Table 2:** Measurable predictors of a favorable response to immune checkpoint inhibitor therapy.

Predictor	Reference
Expression of immune checkpoint inhibitory molecules on intra-tumoral T cells, as well as their ligands on tumor cells	(2, 29)
Expression of immune checkpoint inhibitory molecules on, and their mRNA transcripts in, circulating T cells, as well as serological detection of the soluble forms of these molecules	(30, 34)
Detection of high numbers of total circulating lymphocytes and CD14^+^ monocytes, as well as increased neutrophil:lymphocyte and platelet:lymphocyte ratios and low numbers of eosinophils, measured pre-therapy	(43, 44)
Detection of increasing numbers of total lymphocytes, especially CD4^+^/ICOS^+^ T cells, as well as a decreasing neutrophil:lymphocyte ratio during therapy	Reviewed in Ref. (43)
Low levels of circulating soluble CD25 pre- and during therapy	(43, 45)
High-baseline levels of lactate dehydrogenase	(43)
Elevated pre-therapy serum concentrations of IFN-γ/IL-6/IL-10	(42)
Whole blood gene profiling detection of a 15-gene signature comprised of “predictor” and enhancer genes	(46)
Possible associations with specific HLA alleles	(47)

## Immune-Related Adverse Reactions (IRAEs) Triggered by Negative Immune Checkpoint Molecule-Targeted Immunotherapy

The development of, or worsening of existing autoimmune/inflammatory disorders is the hallmark of immune dysregulation secondary to MAb targeting of inhibitory immune checkpoint molecules in patients with advanced malignant diseases. IRAEs, are commonly encountered and potentially fatal with frequencies of up to 70 and 90% in patients treated with PD-1/PD-L1 and CTLA-4, respectively [reviewed in Ref. (16)]. Almost all organ systems are vulnerable to development of IRAEs, albeit with variable timing of onset and severity, according to types of organ and immune checkpoint inhibitor. In some cases, these may only develop following completion of immunotherapy and tend to be most severe following administration of MAbs which target CTLA-4 (53, 54).

There is currently a somewhat limited literature focused on the immunopathogenesis of IRAEs, as well as on the detection of biomarkers which may be predictive of development of IRAEs (43, 55, 56). As mentioned earlier, IRAEs affect almost all organ systems, most commonly “the skin (pruritus, rash, and vitiligo), the gastrointestinal tract (GIT, enterocolitis), the liver (hepatitis), and the endocrine system” (43), while neurological adverse events appear to occur less frequently (57). In this context, it is noteworthy that although they are equipped with abundant and effective cellular immune defenses, the GIT, liver, lungs, and skin are maintained in an immunologically quiescent, albeit vigilant state, involving, among other mechanisms CTLA-4-expressing Tregs (58), which may explain the vulnerability of these organs for development of IRAEs. These adverse events in relation to anti-CTLA-4 anti-PD-1/-PD-L1 are summarized in Table 3 (59).

**Table 3:** Severe immune-related adverse events associated with anti-CTLA-4 and anti-PD-1/-PDL-1 therapy.

Event	%
Rash (general)^a^	13.4–26.0
Rash (maculo-papular)	1.5–17.5
Pruritis	14.1–35.4
Vitiligo	74.3–11.0
Pneumonitis	0.4–7.8
Colitis	0.5–7.0
Diarrhea	4.1–9.0
Hypothyroidism	1.2–7.0
Hyperthyroidism	0.3–7.1
Adrenal insufficiency	0.2–5.5

*^a^Reviewed in Ref. (59)*.

## Immunopathogenesis of IRAEs

Interference with the homeostatic mechanisms of immunological tolerance during administration of immune checkpoint inhibitors appears to underpin the vulnerability of various organs for development of IRAEs. These result from various immunopathogenetic mechanisms including:

- autoantibody-/complement-mediated
- Th1-dependent, cytokine-driven cytotoxic T cell/macrophage-dependent mechanisms
- Th2-dependent, cytokine-driven recruitment, and activation of eosinophils
- T helper 17 (Th17)-dependent, cytokine-driven recruitment and activation of neutrophils, and monocytes/macrophages
- perforin/inflammasome-driven. In this setting, perforin derived from activated cytotoxic T cells has been reported to activate the NLRP3 inflammasome in antigen-presenting cells, promoting maturation of IL-1β (60)
- combinations of these.

Notwithstanding unmasking or exacerbation of pre-existent, antibody-driven autoimmune disease, there is emerging evidence that the majority of IRAEs triggered by immune checkpoint inhibitor therapy appear to have a cell-mediated immunopathogenesis. With respect to the former, myasthenia gravis, most commonly in cancer patients receiving the PD-1 antagonist, nivolumab (61–63), as well as autoimmune hemolytic anemia in those receiving either nivolumab or ipilimumab (64–67), represent documented examples of predominantly autoantibody-driven IRAEs. On the other hand, the immunopathogenesis of the more commonly occurring immune checkpoint inhibitor-therapy-associated IRAEs, such as enterocolitis in particular, as well as dermatological manifestations, hepatitis, pneumonitis, arthritis, and others, appears to involve T cell-driven inflammatory mechanisms. Although limited, the most informative insights into pathogenesis have originated from studies on the pathogenesis of ipilimumab-associated enterocolitis in patients with advanced melanoma.

The frequent occurrence of enterocolitis during immune checkpoint inhibitor therapy is hardly surprising given that GIT has been estimated to represent up to 80% of the entire human system, with the lamina propria containing most of the lymphocytes (68). Although contentious (69), this magnitude of colonization of the GIT by cells of the adaptive, as well as the innate, immune systems underscores the fact that the gut acts as the major portal of entry to a vast array of antigens of both infective and non-infective origin. Aside from conferring protection against potential pathogens, the gut also plays a major role in conditioning the immune system to tolerate beneficial, commensal bacterial colonists (70, 71). Together with resident CTLA-4-expressing Tregs and other types of immune cells, such as sub-types of dendritic cell and innate lymphoid cells, this benign commensal microbiota contributes to maintaining the GIT in a quiescent, albeit vigilant state (70–72).

Given the importance of CTLA-4-expressing Tregs in maintaining immune homeostasis in the GIT, it is not surprising that therapeutic administration of CTLA-4-targeted MAbs in particular would predispose to development of enterocolitis. In this context, it is noteworthy that the clinical and histopathological presentation of immune checkpoint inhibitor-associated colitis is somewhat comparable with that of an inflammatory bowel disease (IBD) flare (73). This condition is believed to be driven predominantly by Th17 cells and results in a breakdown of tolerance to the commensal microbiota and a consequent influx of inflammatory cells into the gastric mucosa (74–78), which is associated with increased fecal levels of calprotectin, a biomarker of neutrophilic inflammation (78).

Although the association of enterocolitis with immune checkpoint inhibitor therapy in advanced, metastatic cancer is well recognized, relatively few studies appear to have focused on the immunopathogenesis of this condition, and its broader implications for development of IRAEs at other anatomical sites. As mentioned above, those of relevance in this context have mainly focused on ipilimumab therapy of metastatic melanoma. The first and apparently the most comprehensive of these was an early clinical trial reported by Beck et al. in 2006 to which 137 patients with metastatic melanoma were recruited, some of whom (*n* = 56) received both ipilimumab and a melanoma vaccine (79). Forty-one patients (21%) developed colitis, 39 of whom had histological evidence of disease. Three types of colonic cellular infiltrate were evident *viz*. neutrophilic inflammation only (41%), lymphocytic infiltration only (15%), and a combined neutrophilic/lymphocyte infiltrate (38%) (79). The apparent involvement of neutrophilic inflammation in the pathogenesis of ipilimumab-associated enterocolitis is supported by the findings of whole blood gene profiling in patients with this condition, which demonstrated increased expression of genes encoding the neutrophil surface activation markers, CD66a and CD177 (80).

Another early study by Maker et al., to which 36 patients with grade IV melanoma were recruited, investigated the immunotherapeutic potential of ipilimumab in combination with recombinant IL-2 (81). Of the four patients (11.1%) who developed enterocolitis, histopathologic analysis of the colon was performed on three patients, with T cell infiltration documented in two of these, while analysis of the third revealed “crypt destruction, loss of goblet cells, and neutrophilic infiltrates in the crypt epithelium” (80).

In a very recent study, Bamias et al. described the immunological features of ipilimumab-associated colitis in nine patients with advanced melanoma (82). These authors also reported that “endoscopic characteristics resembled IBD and histology revealed predominance of plasmacytes or CD4^+^ T cells” (82). Importantly, the authors detected significant involvement of both the Th1 and Th17 effector pathways according to upregulation of IFN-γ and IL-17A messenger RNA (increases of >10- and 5-fold, respectively, *P* < 0.01), consistent with a T cell driven, pro-inflammatory immunopathogenesis (82). This contention, particularly the involvement of Th17 cell activation in the pathogenesis of CTLA-4-targeted, and possibly other types of immune checkpoint inhibitor immunotherapy, is supported by another study which documented increased circulating levels of IL-17 in patients with metastatic melanoma at 7 and 12 weeks post-initiation of therapy (*P* = 0.007 and *P* = 0.02, respectively) (83). Additional support is derived from the study by Tarhini et al., who reported that elevated baseline levels of circulating IL-17 were significantly (*P* = 0.02) associated with the development of enterocolitis in ipilimumab-treated patients with advanced melanoma (38). The involvement of T cells in driving immune checkpoint inhibitor immunotherapy is also supported by the apparent clinical utility of vedolizumab, an MAb which antagonizes the T cell gut homing receptor, α4β7, in the treatment of immune checkpoint inhibitor-induced enterocolitis (84).

In addition to enterocolitis, IRAEs commonly associated with ipilimumab therapy include hepatitis, endocrinological, and cutaneous disorders, which may be persistent and even fatal, while those associated with anti-PD-1 therapies include thyroid disease and pneumonitis in the case of nivolumab and dermatitis in the case of pembrolizumab (85). As alluded to earlier, the propensity to develop colitis during therapy with ipilimumab is most probably related to suppression of the high numbers of CTLA-4-expressing Tregs in the GIT, unleashing Th17-driven inflammatory responses. Similar mechanisms may underpin the immunopathogenesis of other types of ipilimumab-associated IRAEs such as hepatitis (86) and inflammatory arthritis (87). In addition, excessive expansion of Th17 cells in the GIT, associated with alterations in the gut microbiota, has been reported to exacerbate autoimmune disorders occurring at distal anatomical sites such as multiple sclerosis (88).

In the case of immune checkpoint inhibitor-associated inflammatory arthritis, Cappelli et al. recently reported on the occurrence of this IRAE in nine patients with various types of advanced malignancy treated with either the combination of ipilimumab and nivolumab (*n* = 7) or with nivolumab only (*n* = 2) (87). Four patients receiving combination therapy also developed colitis, which preceded the arthritis in three patients. Serological analysis failed to reveal the presence of classical RA-associated autoantibodies (rheumatoid factor and anti-citrullinated peptide/protein antibodies), while synovial fluid analysis performed on four patients revealed a predominantly neutrophil inflammatory infiltrate (87). The authors speculated that “the large joint involvement in most patients, along with the reactive arthritis phenotype and coexisting colitis, suggest a possible Th17-mediated mechanism of inflammatory arthritis” (87). While supporting evidence for this contention exists in the case of ipilimumab, it is noteworthy that antagonism of PD-1 has also been reported to promote Th1- and Th17-mediated immune responses. In this context, human RA synovium and synovial fluid have been reported to be “enriched” with PD-1-expressing T cells, while in a murine model of experimental arthritis, PD-1 gene knockout mice (*PD-1^−/−^*) demonstrated increased susceptibility for development of collagen-induced arthritis, which was associated with increased T cell proliferation and production of IFN-γ and IL-17 (89). In addition, treatment of whole blood or isolated mononuclear cells from patients with prostate cancer or melanoma with a PD-1-targeted MAb, followed by activation of T cells, resulted in a pro-inflammatory response characterized by enhanced production of IL-2, IL-6, IL-17, TNF-α, and IFN-γ and reduced production of the Th2 cytokines, IL-5, and IL-13 (90).

Taken together, the aforementioned findings suggest that the immunopathogenesis of CTLA-4- and PD-1-inhibitory therapy-associated IRAEs is most commonly driven by Th1- and Th17-dependent inflammatory mechanisms. This contention is supported by the apparent plasticity of Th1/Th17 cells in the immunopathogenesis of autoimmune disorders. For example, in a murine model of Th17 cell-mediated experimental autoimmune encephalomyelitis, it was observed that exposure of Th17 cells to IL-23 resulted in the generation of dual IL-17^+^/IFN-γ^+^- expressing T cells (91). Moreover, in a murine model of CD4^+^ T cell adoptive transfer-mediated colitis, this condition was found to be associated with Th17 cell reactivity against the commensal enteric microbiota, while disease pathogenesis resulted not only from Th17 cell-dependent mechanisms, but also involved transitioning of Th17 cells to Th1-like cells with an IL^−^17^−^/IFN-γ^+^ phenotype, as well as supporting the development of classical Th1 cells (92).

**Figure 1:**
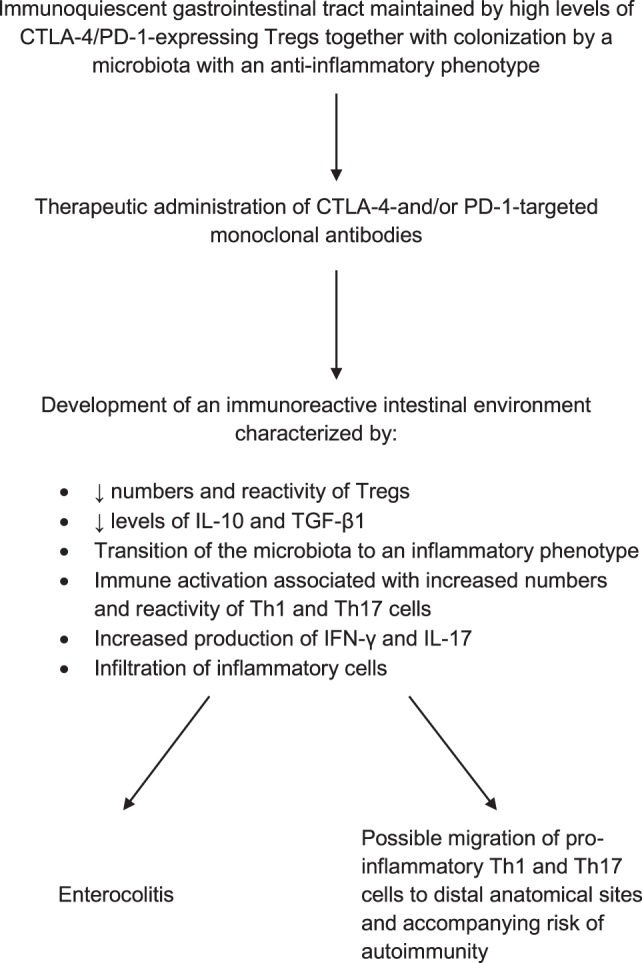
Proposed mechanism of immune checkpoint inhibitor therapy-associated enterocolitis and its possible involvement in the pathogenesis of adverse immune/inflammatory events at distal anatomical sites.

Although remaining to be conclusively established, Th17 cells in particular may have a key involvement in the pathogenesis of immune checkpoint inhibitor-mediated IRAEs. Potential mechanisms of immunopathogenesis are summarized in Figure 1.

## Biomarker-Based Strategies to Reduce the Risk of Development of IRAEs During Immune Checkpoint Inhibitor-Targeted Therapy

Two major approaches fall into this category. Firstly, as described above, strategies that may enable the early identification of those cancer patients who are most likely to experience greatest benefit from immune checkpoint inhibitor-targeted therapy, which, in turn, may enable reductions in the dose and/or duration of therapy. Secondly, the identification of potential biomarkers measured prior to, as well as during, the course of immunotherapy, which may enable early recognition of those cancer patients at highest risk for development of IRAEs. It is this second approach which represents the major focus of the final section of this review. It does not, however, include considerations of the possible utility of autoantibody panels, or other current recommendations in respect of the pre-immunotherapy work-up and subsequent monitoring strategies in patients with advanced cancer, both of which have been described in detail elsewhere (16, 56). Instead, it is focused on largely unexplored strategies, some of which may have predictive potential.

### Cytokine Profiles and C-Reactive Protein

Measurement of the concentrations of circulating inflammatory and anti-inflammatory mediators may be predictive of development of immune checkpoint inhibitor therapy-associated IRAEs. This contention is based on observations that increased concentrations of circulating pro-inflammatory, as well as, counteracting anti-inflammatory cytokines precede the onset of clinically evident RA, most prominently in patients with seropositive disease (93, 94). In a study reported by Kokkonen et al., increases in the concentrations of IL-1β, IL-2, IL-6 and those indicative of activation of Th1 cells, specifically IFN-γ and IL-12, together with those of a co-existent anti-inflammatory response (IL-10 and IL-1 receptor antagonist) were found to pre-date the onset of clinically evident RA (93). In addition, levels of IL-17 were highest in IgM-rheumatoid factor-seropositive, pre-RA patients, declining following the onset of disease, leading the authors to propose that the role of IL-17 is most significant in the initiation phase of RA (93). These findings were largely confirmed by Deane et al., who did not, however, include IL-17 in their profile of cytokines/chemokines (94). These authors also reported that elevated levels of CRP were predictive of development of RA (94).

Together with the findings alluded to earlier in this review that elevated levels of IL-17 and IFN-γ measured prior to, as well as subsequent to, administration of ipilimumab are associated with the development of enterocolitis (38, 82, 83), it is possible that combining these two cytokines with a limited selection of additional biomarkers may enhance prediction of development of this and other IRAEs when measured pre-therapy. Potential additional biomarkers for inclusion in this screening panel include CRP, as already recommended by Kostine et al. (56) and supported by the findings of Rastogi et al. in patients with ipilimumab-associated enterocolitis (95), together with IL-10 and TGF-β1 (38).

In summary, a biomarker profile incorporating IL-17/IFN-γ/IL-10/TGF-β1/CRP appears to merit evaluation in the prediction of susceptibility for development of IRAEs.

### Biomarkers of Systemic Activation of Neutrophils

The findings alluded to above of increased fecal levels of the neutrophil-derived biomarker, calprotectin (78), albeit in IBD, as well as elevated levels of mRNA transcripts encoding the neutrophil surface markers, CD66 and CD177 (80), in patients with ipilimumab therapy-associated enterocolitis, suggests that the presence of elevated concentrations of circulating biomarkers of neutrophil activation may be predictive of development of IRAEs. Although not specific for neutrophils, the primary and tertiary granule enzymes, myeloperoxidase and matrix metalloproteinase 9, respectively, as well as the soluble form of the cell surface adhesin, L-selectin, are biomarkers which may merit evaluation in this context, possibly complemented by flow cytometric detection of upregulated expression of surface biomarkers associated with neutrophil activation.

### Cotinine

Although smoking is a predictor of a favorable response to PD-1 targeted immunotherapy in patients with non-small cell lung carcinoma (29, 51), it may also be associated with susceptibility for development of IRAEs. This contention is based on four different lines of evidence. These are: (i) active smoking is associated with increased levels of circulating and tissue levels of IL-17 in humans (96, 97), as well as with increased numbers of circulating levels of Th17 cells in a murine model of exposure to cigarette smoke (98); (ii) active smoking is associated with increased numbers and pro-inflammatory activity of circulating neutrophils (99–101); (iii) cured tobacco contains many different types of potentially pro-inflammatory microorganisms (102), which may explain in part the adverse effects of smoking on the composition of the microbiota of the GIT (103); and (iv) smoking is associated with predisposition for development of various types of autoimmune disease (104–106). Objective assessment of exposure to cigarette smoke, and possibly consumption of smokeless tobacco products, may therefore be predictive of susceptibility for development of IRAEs. In this context, measurement of the nicotine metabolite, cotinine, in blood or urine may be a useful predictive strategy as levels of this biomarker of tobacco exposure are significantly elevated in active smokers and those exposed to sidestream smoke, as well as in users of smokeless tobacco products (107).

## Gut Microbiome

Alterations in the gut microbiome due to factors such as diet, smoking, and co-existent intestinal inflammation appear to cause decreases in the numbers of quiescent commensal microorganisms in the GIT, such as those belonging to the *Firmicutes* and *Actinobacteria* families (70, 88, 103). This is likely to result in expansion of pro-inflammatory Th17 cells, which may sensitize the GIT to immune checkpoint inhibitors, particularly those which target CTLA-4 as mentioned above. The likely consequence is a predisposition for development of enterocolitis and possibly other IRAEs due to the trafficking of Th17 cells to distal anatomical sites (88). Pre- and post-therapy GIT microbiome profiling using 16S ribosomal sequencing of fecal samples, may therefore enable identification of patients at risk for development of IRAEs.

## Microbial Translocation

Although also largely unexplored, immune checkpoint inhibitor-mediated dysregulation of Treg cell function and expansion of Th1 and Th17 cells in the GIT, together with the associated transition of the gut microbiota to a more inflammatory milieu may favor development of microbial translocation. This process, which is common in HIV-infected patients, occurs as a consequence of inflammation-mediated damage to the intestinal mucosa, resulting in leakage of pro-inflammatory microbial products into the systemic circulation (108). These bacterial-derived agents, such as lipopolysaccharide and nucleic acid, promote low-grade systemic inflammation, largely caused by activation of Toll-like receptors expressed on monocytes/macrophages (108). Microbial translocation, in turn, may predispose to development of IRAEs. Circulating biomarkers which are associated with microbial translocation include intestinal fatty acid-binding protein and zonulin, which are markers of increased epithelial permeability, as well as lipopolysaccharide-binding protein, soluble CD14 and CD163 (109–111), which are indicative of the presence of bacterial lipopolysaccharide in the systemic circulation. Detection of increased levels of these biomarkers prior to and during immune checkpoint inhibitor therapy may predict development of enterocolitis and possibly other IRAEs.

Biomarkers reviewed in this section which may be predictive of development of IRAEs are summarized in Table 4.

**Table 4:** Putative strategies to predict of development of immune-related adverse events (IRAEs) during immune checkpoint inhibitor therapy.

Putative strategy	Reference
Application of a cytokine/CRP-based circulating biomarker profile consisting of IL-17/IFN-γ/IL-10/TGF-β1/CRP measured prior to and during therapy	(38, 82, 83, 93–95)
Measurement of a limited number of circulating biomarkers of neutrophil activation, e.g., myeloperoxidase/matrix metalloproteinase 9/L-selectin/others	(78, 80)
Measurement of cotinine in blood or urine as an objective indicator of tobacco usage and associated systemic inflammation and pro-inflammatory changes in the gut microbiota, which may favor development of IRAEs	(96–107)
Detection of alterations in the gut microbiota consistent with the transition to a pro-inflammatory phenotype conducive to development of IRAEs	(70, 88, 103)
Measurement of systemic biomarkers of microbial translocation indicative of inflammation-mediated damage to the intestinal mucosa and resultant low-grade systemic inflammation. Biomarkers in this category include intestinal fatty acid-binding protein, zonulin, lipopolysaccharide-binding protein, soluble CD14, and soluble CD163	(107–111)

## Conclusion

Although its promise is incompletely fulfilled, immune checkpoint inhibitor-based immunotherapy of cancer remains an innovative and challenging branch of oncology which is likely to experience significant advances in the foreseeable future. This contention is based on the probable increase in therapeutic options due to an expanding repertoire of therapeutic MAbs targeting novel immune checkpoint inhibitory molecules, which may be used as monotherapy or, more likely, in combination therapy. Therapeutic efficacy may be further improved by the realization of strategies which enhance tumor neoantigenicity, together with the identification of biomarker-based approaches predictive of favorable responses to therapy. Finally, insights into the predominantly Th17-driven inflammatory mechanisms implicated in the immunopathogenesis of IRAEs, mainly ipilimumab-associated enterocolits, may enable identification of biomarker-based strategies, albeit largely unexplored, which may be predictive of the development of IRAEs.

## Author Contributions

Both authors contributed significantly to the design and content of the manuscript.

## Conflict of Interest Statement

The authors declare that the research was conducted in the absence of any commercial or financial relationships that could be construed as a potential conflict of interest.
